# Investigating significant health trends in growth hormone treatments registry: rationale, aims and design of a nationwide prospective registry (study protocol)

**DOI:** 10.1186/s13023-023-02716-3

**Published:** 2023-05-10

**Authors:** Dirk Schnabel, Ilonka Kreitschmann-Andermahr, Christian J. Strasburger, David Pittrow, Christine Pausch, Joachim Woelfle, for the INSIGHTS-GHT Study Group

**Affiliations:** 1 Pediatric Endocrinology, Center for Chronically Sick Children, Charité Universitaetsmedizin, Berlin, Germany; 2 Department of Neurosurgery and Spine Surgery, University Medicine Essen, University of Duisburg-Essen, Essen, Germany; 3 Department of Medicine for Endocrinology, Diabetes and Nutritional Medicine, Charité Universitaetsmedizin, Campus Mitte, Berlin, Germany; 4 Institute for Clinical Pharmacology, Medical Faculty, Technical University, Dresden, Germany; 5 Innovation Center Real-World Evidence, GWT-TUD GmbH, Dresden, Germany; 6 Department of Pediatric and Adolescent Medicine, Friedrich-Alexander-University, Erlangen, Germany

**Keywords:** Growth hormone deficiency, Human growth hormone, Somatropin, Adults, Children, Observational

## Abstract

**Background:**

Somatropin treatment is indicated in a variety of disorders including growth hormone (GH) deficiency, Prader–Willi and Turner syndrome, chronic renal insufficiency and others. To date, almost all studies have been limited to single GH products, and no independent registry across indications and somatropin products was ever established.

**Aim:**

The present investigator-initiated registry named INSIGHTS-GHT aims to provide comprehensive information on various aspects of somatropin treatment in Germany in approved indications within routine clinical practice: drug utilization, effectiveness (including real final height, body composition), tolerability, quality of life, other patient related outcomes (PRO), and health economic variables.

**Methods:**

Registry (prospective observational study) in specialised pediatric and adult endocrinology centres in Germany. Patients of any age are eligible for documentation, if they are on ongoing or newly initiated treatment with any approved somatropin or somatropin-related product within the labelling, available for long term follow-up documentation, and if they provided informed consent. Subjects may switch, discontinue/interrupt or initiate somatropin products at any time. They are followed up for at least 3 years (minimal study duration). Documentation is planned once or twice per year to record somatropin utilisation (product, dosing), other medications, laboratory status (glucose, lipids, GH function including stimulation tests, IGF-I, IGFBP3), if applicable, pubertal development, auxological parameters, body composition and bone age. Patient reported outcome (PRO) measures include, but are not limited to, Short Form 12 in adults and adolescents aged 14 years and over. Safety reporting includes adverse events.

**Conclusions:**

The registry documents children and adults in one joint registry, includes, at present, patients in Germany and allows documentation of patients on all approved somatropin and other growth hormone preparations. It will allow to describe the transition of subjects from adolescence to adulthood (treatment and height), to describe switches between somatotropin preparations, to perform responder analyses, and to analyse differences and similarities of somatropin utilization (by age group, sex, setting, and PRO instrument). INSIGHTS-GHT offers a broad, comprehensive research platform to assess multiple relevant aspects of somatropin treatment and outcomes (including the transition of subjects from adolescence to adulthood), allows the documentation of all GH products including long-acting GH preparations after their introduction, and will evaluate the data independently of funders.

*Trial registration* BfArM Nr. NIS7492, DRKS registry DRKS00027394.

## Background

Growth hormone (GH), Secreted by the anterior pituitary gland, plays a pivotal role in growth and metabolism.

In pediatric patients (infants, children and adolescents) growth hormone deficiency (GHD) is a rare condition that may occur due to various causes. Subdivisions of GHD include isolated and multiple pituitary hormone deficiency, congenital (present from birth), acquired (e.g., due to tumour, result of radiation damage, infection, trauma), and idiopathic forms (no known cause). The conditions may result in infantile hypoglycaemia, growth retardation and short stature, and maturation delays reflected by the delay of lengthening of the bones of the extremities that is inappropriate to the chronological age of the child. There is also a prescribability for supraphysiological non-replacement GH for some other indications with short stature. The term “small for gestational age” (SGA) generally describes any infant whose birth weight and/or length is less than -2 SDS (standard deviation score, related to gestational age). Prerequisites for GH therapy for these children include a lack of spontaneous catch-up growth until the 4th birthday, a body height below 2.5 standard deviations (− 2.5 SDS) and a height velocity SDS for chronological age below zero (HV-SDS < 0). Turner syndrome is a sex-chromosomal disorder affecting 1 of 2500 females and is frequently characterized by short stature and additionally in most cases by the lack of spontaneous puberty. Prader–Willi syndrome (PWS) is a genetic disorder characterized by low muscle tone, short stature, incomplete sexual development, and a chronic feeling of hunger that, coupled with a metabolism that utilizes fewer calories than normal, can lead to excessive eating and life-threatening obesity [1]. Growth disorders in chronic renal insufficiency (CRI) also may require GH treatment (GHT). Short stature in PWS and CRI results, among other possible causes, from abnormalities of the GH–IGF axis.

In adults, GHD is associated with increased visceral adiposity, decreased lean body mass, bone mineral density and exercise capacity, dyslipidaemia, insulin resistance, increased cardiometabolic and fracture risk, and impaired quality of life [2]. Adult GHD can persist from childhood, or occurs due to structural lesions such as pituitary adenomas and other tumours of the sellar region, radiotherapy to the pituitary, traumatic brain injury and other rare causes [3].

Typical treatment of GHD due to the named conditions in children and adults is daily injection of recombinant GH. A number of products have been licensed in various indications. Furthermore, since 2022 EMA has approved long-acting GH preparations for the treatment of GHD. A large body of evidence has been gathered on the efficacy/effectiveness and safety of these substances, both from randomized controlled trials and from observational research [4–9]. Yet, there are many open research questions that can only or preferably be addressed by further observational research.

## Aims

The present registry aims to provide comprehensive information on different aspects of somatropin treatment in Germany within routine clinical practice: utilization, efficacy, safety, quality of life and other patient related outcomes (PRO), and economic variables, including post-treatment follow-up in various approved indications.

The registry documents children and adults in one joint registry, includes, at present, patients in Germany and allows documentation of patients on all approved somatropin and other growth hormone preparations.

It will allow to describe the transition of subjects from adolescence to adulthood (treatment and height), to describe switches between somatotropin preparations, to perform responder analyses, and to analyse differences and similarities of somatropin utilization (by age group, sex, setting, and PRO instrument).

The newly initiated registry INSIGHTS-GHT can document all indications for GH therapy in the context of the introduction of new therapies and complement the data from randomised controlled trials. It may serve quality assurance purposes, as individual centres can compare their results with other centres and with guideline recommendations.

## Methods

### Objectives and endpoints

The objectives and corresponding endpoints of the registry are shown in Table 1.

**Table 1:** Objectives and endpoints

	**Objective**	**Endpoints**
**Primary**	To analyse somatropin drug utilization within routine clinical practice in a diverse population of children and adults, across all GH products To determine the place of long-acting GH in practice	Utilized GH preparations Dose and dosing schedule (daily vs. long-acting) Duration of treatment Persistence Switches between GH treatments Interruption/ discontinuation of GH treatment
**Secondary**	**Efficacy** To describe the long-term efficacy of somatropin To assess the transition from adolescence to adulthood	Paediatric indications: Real final adult height (using stadiometer) Adult indication: Body composition (all methods) Delta IGF-I standard deviation score (SDS, change during treatment / normalization) Delta height standard deviation score (SDS) / delta lean body mass Body proportions (sitting height; standing height), Delta target height vs height SDS Lipid status, inflammation markers, IGF-I, blood pressure Analyses stratified according to different diagnosis groups
	Persistence	Persistence calculated as duration of time from initiation to discontinuation of therapy
	Patient related outcomes (PRO) To describe patient PRO, including quality of life and patient experience over the initial 2 years of GH treatment, cross-sectional and long term (children and adults)	Satisfaction with treatment experience Quality of life, e.g., by SF-12 (in adults and adolescents) Additional validated self-reported or observed (e.g., parent- or partner-rated) QoL and PRO questionnaires can optionally be used
	**Safety** To describe the long-term safety of somatropin	Number and rate per patient-years of Adverse events All causally related and/or temporally associated AEs
**Exploratory**	Exploratory: To assess / analyse additional parameters potentially modulated by GHT To assess subgroups of the population under observation, or to assess outcomes by participating centres	e.g., body composition by DXA

### Ethical aspects and data protection

#### Ethical approval

The registry is conducted consistent with consensus ethics principles derived from international ethics guidelines including the Declaration of Helsinki, and applicable local regulatory requirements and laws.

Prior to any data collection under this protocol, written informed consent with the approved ICF is obtained from the patient or patient’s legally authorized representative, as applicable, in accordance with local practice and regulations.

Detailed information about the registry and that registry participation is voluntary will be explained to the patient. This information may be provided in a personal conversation, and alternatively via telephone or videoconference. The patient will be given sufficient time to consider whether to participate in the registry. The signed ICF will be retained with the registry records. A copy of the ICF, signed and dated by the patient, must be given to the patient.

The patient or its guardian can revoke registry participation at any time. Patients who have revoked their participation will not be replaced.

### Confidentiality and data protection

The sponsor as well as all investigators ensure adherence to applicable data privacy protection regulation. Data are transferred in encoded form only. The entire documentation made available to the sponsor does not contain any data which, on its own account or in conjunction with other freely available data, can be used to re-identify natural persons. The investigators are obligated to ensure that no documents contain such data.

All records identifying the subject will be kept confidential and will not be made publicly available. Patient names will not be supplied to the sponsor. If the patient’s name appears on any document, it must be obliterated before a copy of the document is supplied to the sponsor. Registry findings stored on a computer will be stored in accordance with local data protection laws.

The investigator will maintain a list to enable patients’ records to be identified in case of queries. In case of a report of a serious adverse drug reaction (SADR), the responsible pharmacovigilance person may ask for additional clarification. In that case, the company is not allowed to directly contact the patient. All additional information will be provided by the investigator.

Data protection rules will be closely followed and the standards of the General Data Protection Regulation (GDPR) will be met. Neither initials nor the exact birth date of the patient will be recorded in the database. Patient data are collected under a pseudonym. Upon saving of the first assessment (= inclusion visit), the EDC system automatically assigns a unique and consecutive Subject Identification Code (SIC). All registry documents (e.g., print outs of electronic case report forms, the informed consent forms etc. are identified with the SIC. The SIC alone cannot be used for identification of a given patient outside a site, in compliance with laws governing data privacy.

## Design

INSIGHTS-GHT is a post-marketing, multicentre, prospective, non-controlled, non-interventional registry. It allows for structured, non-interventional collection of data.

### Observational plan

Participating physicians will not be subject to any instructions regarding the diagnosis and therapy of their patients. All examinations performed depend on the discretion and clinical routine of the physician.

The registry contains a basic set of variables (mandatory data) which are essential for all included patients. Another set of variables (facultative data) can be requested from the participating sites but is left to their discretion. The platform is designed to accommodate substudies (i.e., addition of further variables) in selected centres to facilitate research collaboration between institutions.

### Setting

This registry will be performed in approximately 60 specialized endocrinology sites in Germany that have expertise in growth hormone therapy. Any decision to prescribe these products will be made at the discretion of the treating physician and independent from and before any participation in this registry.

### Characteristics of patients

The eligible registry population will consist of children and adults of any age and of all sexes (male, female, diverse).

There is no formal screening procedure for suitable patients. Physicians are requested to assess all patients receiving somatropin products (already treated or newly to be initiated) for potential suitability for inclusion in the registry.

Patients are eligible for documentation if they.

- Are treated with any approved somatropin product,
- Are treated within the labelling of the respective product,
- Are available for long term follow-up documentation,
- (Or their parents/guardians, respectively) have provided informed consent.

Patients may change the somatropin product at any time, also repeatedly, during the course of documentation.

Patients are not eligible if they concurrently participate in a GH-related controlled clinical trial.

## Processes

### Treatment

Any approved somatropin product may be documented during the registry if used within its respective labelling at the time of documentation. In the protocol, the product names or manufacturers are not specified as during the course of the registry the availability of products or their labelling may change.

Detailed conditions for the use of the somatropin products and contraindications, special warnings and precautions for use are described in accordance with the marketing authorization in the Summary of Product Characteristics (SmPC) and/or the prescribing information.

### Interruption or discontinuation of GHT

Once included in the registry, it is anticipated that children will be treated with somatropin until achieving final height.

However, somatropin treatment may be interrupted or prematurely discontinued. Patients who interrupt GHT for any reason may remain in the registry to be further documented, or if they resume GHT at a later stage, continue documentation under the original patient number.

Every patient has the right to withdraw his/her Informed Consent for the surveillance at any time without giving any reason. All data generated up to the time of withdrawal of the Informed Consent or discontinuation from the surveillance including all periods of interruption of somatropin treatment will be analysed and the reason(s) for each interruption and the definite discontinuation will be recorded.

### Concomitant medication

All concomitant medications will be documented with generic drug name (INN) or trade name, with start and stop dates.

### Visit schedule and assessments

The frequency of visits is at the discretion of the treating physician. It is expected that for each patient 1 to 2 visits per year are documented. At each visit, relevant information since the last documentation is collected.

Documentation of data from visits can be performed onsite or remotely (e.g., via telephone or video chat), however such data must be part of the patient charts (exception: data from PRO questionnaires). For the documentation, the following forms are available: inclusion visit, follow-up visit, former medication, current medication, other (non-GH) medication, adverse events.

Owing to the observational study type, no additional or specific visits, tests or assessments are required for the purposes of this registry (exception: PRO questionnaires).

### Inclusion Visit

At the Inclusion visit, the following data will be recorded, if available.^1^

- Date of Informed Consent (mandatory)
- Date of visit/ contact with patient
- Demographics: year of birth, month of birth*, sex, ethnicity
- Physical examination: height, sitting height, weight, systolic and diastolic blood pressure, heart rate, head circumference
- Previous participation in other GH databases (registries, non-interventional studies (NIS), other): NIS/registry name, if available patient ID
- Previous or current participation in a clinical trial (which does not investigate GH products): start/stop date, indication

Indication for GHT: date of diagnosis, diagnosis.

*Parents’ height, height and weight at start of GH therapy.

*Birth history: Gestational age, birth weight and length, head circumference.

*Pubertal development: Tanner stages (pubic hair, breast development, male genitals); testicular volume in males, start date of puberty, spontaneous/induced puberty, menarche in females.

*Bone age: method, dates and results of current and previous (before start of GHT) evaluations.

Bone density: method, dates and results of current and previous (before start of GHT) evaluations.

Body composition: method (DXA or impedance) and device for measurement; dates and results for fat mass, lean body mass, total body water.

Prior and concomitant diseases (including information on scoliosis).

Patient-related outcomes: Short Form 12 (in adults and adolescents aged 14 years and over).

Former GHT: product name, start/stop date, reason for discontinuation.

Current GHT: product name, formulation and strength, body weight at first dose, start/stop date.

Concomitant medication: generic name or trade name, start/stop dates.

Laboratory results before start of GHT (value, date and assessment, if not otherwise specified).

- Glucose metabolism: HbA_1C_
- Lipid status: LDL cholesterol, HDL cholesterol
- Hormone analysis (date, assessment and substitution): thyroid axis, gonadal axis, adrenal axis, pituitary axis
- Results of GH stimulation tests: Arginine Infusion Test,*Clonidine Test (paediatric only), Insulin Hypoglycaemia Test (IHT), GHRH-Arginine Test, other tests
- IGF-I and IGFBP-3 (value, date, assessment, SDS, normal range, assay)

Laboratory values: value, date and assessment

- IGF-I and IGFBP-3 (value, date, assessment, SDS, normal range, assay)
- Glucose metabolism: glucose, insulin, HbA_1C_
- Inflammation parameter: C-reactive protein
- Lipid status: LDL cholesterol, HDL cholesterol

Function of other pituitary axes (thyroid-, gonadal- and adrenal axis, AVP deficiency): sufficient/insufficient, adequately replaced if insufficient?

Genetics, morphology, additional pituitary hormone deficiencies.

### Follow-up documentations

During the follow-up periods, information will be collected during visits of the patient or via telephone or video contacts at intervals corresponding to routine clinical practice (usually once or twice yearly).

Physical examination: height, sitting height, weight, systolic and diastolic blood pressure, heart rate, *head circumference.

Current participation in a clinical trial: start/stop date, indication.

*Pubertal development: Tanner stages (pubic hair, breast development, male genitals); testicular volume in males, start date of puberty,,spontaneous/induced puberty, menarche in females.

*Bone age: method, date and result.

Bone density: method, date and result.

Body composition: method (DXA or impedance) and device for measurement; date and results for fat mass, lean body mass, total body water.

New and resolved concomitant diseases: with start/stop date.

Patient-related outcomes: i.e., SF-12 in adults and adolescents aged 14 years and over.

Current GHT: product name, formulation and strength, body weight at first dose, start/stop date, if applicable, reason for discontinuation.

Concomitant medication: generic or trade name, start/stop dates.

Laboratory values: value, date and assessment.

IGF-I and IGFBP-3 (value, date, assessment, SDS, normal range, assay).

Glucose metabolism: glucose, insulin, HbA_1C_.

Inflammation parameter: C-reactive protein. 

Lipid status: LDL cholesterol, HDL cholesterol.

Function of other pituitary axes (thyroid-, gonadal- and adrenal axis, AVP deficiency): sufficient/insufficient, adequately replaced if insufficient?

Quality of life (QoL) will be assessed with a validated generic QoL instrument (SF-12). Other instruments/ questionnaires may be added during the course of the registry or be used for specific cross-sectional evaluations.

### Subject completion

Subject may be followed up in the registry until the final registry termination. There is a planned minimum follow-up period of 3 years, but no maximum follow-up period. Interruptions of GHT are acceptable, and in such cases the patient can remain in the registry. Switches from one to another GH preparation does not qualify as subject completion, i.e., patients can remain in the registry.

### Subject discontinuation

Reasons for discontinuation of GHT will be reported as on a follow-up documentation form (end-of-observation visit, EOO). Reasons include end of GHT, withdrawal of informed consent, change of centre (not participating in INSIGHTS-GHT), lost to follow-up or any other reasons, e.g. administrative.

In case of end of the INSIGHTS-GHT registry, the information, whether the final height is reached and if yes, whether it is the genetic target height, is captured.

In case of lost-to-follow up, efforts should be made to contact the patient (by phone or other channels) in order to collect missing information.

Regardless of the reason, all data available for the subject up to the time of discontinuation should be recorded on the appropriate eCRF. Data collected up to the time of discontinuation will be used in the analysis and included in the clinical registry report.

### Safety reporting

The following safety data must be reported, if any of these events occur in patients treated with a GH product:

- Serious Adverse Events (SAE)
- Non-serious Adverse Event (AE)
- Reports of drug exposure during pregnancy
- Reports of misuse and abuse of products
- Reports describing the occurrence of other “special circumstances” and “Special scenarios” (irrespective of whether a clinical event has occurred)

AEs are defined as follows: [10]

An untoward medical occurrence after exposure to a medicine, which is not necessarily caused by that medicine. An AE can therefore be any unfavourable and unintended sign (including an abnormal laboratory finding, for example), symptom, or disease temporally associated with the use of a medicinal product, whether or not considered related to the medicinal product.

SAEs are defined as follows: [10]

Any AE or experience that, at any dose,

- Results in death
- Is life-threatening
- Requires hospitalization or prolongation of existing hospitalization
- Results in permanent or serious disability/incapacity
- Is a congenital anomaly/birth defect
- Is a medically significant

Medical and scientific judgement should be exercised in deciding whether expedited reporting is appropriate in other situations, such as medically significant events that may not be immediately life-threatening or result in death or hospitalization but may jeopardize the patient or may require intervention to prevent one of the other outcomes listed in the definition above. These should also usually be considered serious.

Definition special scenario:

Includes reports of drug-drug/drug-food interaction, drug use during lactation or breast-feeding, lack of effectiveness, overdose, drug maladministration or accidental exposure, dispensing errors/medication errors, withdrawal or rebound symptoms, irrespective of whether a clinical event has occurred.

“Special Circumstances” mean drug exposure during pregnancy, drug misuse and abuse.

Pregnancies in women receiving GHT must be reported in the same way as AEs. The investigator will follow-up pregnancies to determine outcome (including premature termination) and status of mother and child and report it.

### Reporting channels and timelines

The above-described events and pregnancies will be reported by the electronic data entry form on the website of the INSIGHTS-GHT registry. The investigator will be required to describe the event (type, start and stop dates, severity, seriousness, outcomes etc.). Other relevant information from the current and previous documentations will be automatically added to the report. When the investigator saves the AE form in the database, it will be sent via e-mail to the respective marketing authorization holder (as indicated in the current Summary of Product Characteristics) of the GHT, and in copy to GWT-TUD and the investigator himself/herself (as confirmation).

In case the electronic system should not be accessible, the investigator shall alternatively use paper sheets which should be sent via fax to the respective marketing authorization holder of the GHT.

SAE must be reported within 1 business day after knowledge.

Non-serious AE (e.g., all AE that do not meet the definition of serious) should be reported within 14 calendar days coming to the investigator’s attention.

## Assessments

### Specification of efficacy parameters

#### Auxological parameters

The following growth parameters have been found to be meaningful to assess the efficacy of any treatment of patients with growth failure:

- Height Velocity (HV, cm/year) and HV Standard Deviation Score (HV SDS).
- Height Standard Deviation Score (H SDS).

Measurements of height should be preferably done with a stadiometer with 0.1 cm accuracy.

### Growth hormone parameters IGF-I and IGFBP-3

All IGF-I and IGFBP-3 values should preferentially be expressed as standard deviation score for age and the analytical method should be specified.

### BMI and body composition

Children: An improvement of body mass index (BMI) compared to baseline is expected.

Body composition (total fat mass, lean body mass, total body water) may be measured by e.g., Bioelectronical Impedance Analysis (BIA) or Dual Energy X-ray Absorptiometry (DXA) as assessed in clinical routine.

### Patient related outcomes

Patient related outcomes (PRO) capture a person’s perception of their own health through questionnaires.

PRO scales such as the Short Form 12 and others will be administered, either in a longitudinal design or at a given point of time in selected groups in a cross-sectional analysis.

### Short form 12 (in patients aged 14 years and over)

The SF-12 is a health-related QoL questionnaire consists of a subset of twelve questions, derived from the Short Form 36 (SF-36) questionnaire, that measures eight health domains to assess physical and mental health [11]. Physical health-related domains include General Health, Physical Functioning, Role Physical, and Body Pain. Mental health-related scales include Vitality, Social Functioning, Role Emotional, and Mental Health [12].

The self-administered version of SF-12 will be used. A physical component score (PCS) and mental component score (MCS) can be calculated. Country-specific weights are available for various countries including Germany.

### Safety assessments

Safety assessments will consist of all adverse events (overall, and all causally related and/or temporally associated AEs).

### Data collection and quality control

The Sponsor is responsible for implementing and maintaining a quality management system with written development procedures and functional area standard operating procedures (SOPs) to ensure that studies are conducted and data are generated, documented, and reported in compliance with the protocol, accepted standards of Good Epidemiological Practice (GEP) [13], and all applicable laws, rules and regulations relating to the conduct of the registry [14].

Before registry start at the sites, all investigators are sufficiently trained on the background and objectives of the study and ethical as well as regulatory obligations.

Data are collected by the centres online, using a secure connection (no paper–pencil CRFs are be used). The electronic data capture system (EDC) with electronic data collection forms (eCRFs) and other features (e.g., repository of registry documents) is provided by GWT-TUD. No software applications need to be installed by the sites. The web-based EDC application is password protected. User account requests are authorized by GWT-TUD. A manual with instructions how to use the EDC has been made available to sites.

The data are entered by the investigator or registry site personnel via a secure internet connection into the registry database. Only these persons can record or change data relating to patients in their centre on the CRFs.

Detailed information on checks for completeness, accuracy, plausibility and validity are given in the Data Management Plan (DMP). The same plan specifies measures for handling of missing data and permissible clarifications.

Upon entry, the data is automatically checked for completeness and plausibility.

As part of the statistical processing, the data is checked again and queries are sent to the centre if necessary. If needed, requests are sent to the sites to correct incorrect inaccurate data or complete missing data.

In about 10% of the centres, an audit will be carried out to check the correct data entry while respecting data protection (monitoring with comparison of the registry data with original patient data).

## Statistical analysis

### Sample size

As the present registry has mainly descriptive aims, there is no formal power calculation needed to determine the target sample size [15]. Rather, the sample size is determined by feasibility aspects.

Based on outcomes of a recent international registry-type observational study with a cohort of 83.803 patients, it is expected that 56.9% of patients will have different causes of GH deficiency, 9.5% small for gestational age (SGA), 9.2% Turner syndrome, 2.8% Prader–Willi-syndrome, 2.9% chronic renal insufficiency and 18.7% will have other conditions treated with GHT [16].

From a drug safety perspective, an enrolled number of 1500 patients according to the “rule of three” will allow for the detection of AE occurring at an incidence of 1/500 person-years [17, 18]. If the median observation duration is 2.5 years, an AE occurring with an incidence of 1/1250 person-years will be detected.

### Descriptive analysis and statistical testing

Data will be analysed in regular (e.g., six months) intervals. This will be done to keep sites appraised of the progress of the registry and to review data quality (rate of missing values; data distribution, outliers).

All background variables and outcome parameters will be analysed descriptively with appropriate statistical methods: categorical variables by frequency tables (absolute and relative frequencies) and continuous variables by summary statistics (i.e., mean, standard deviation, minimum, median, quartiles and maximum). Continuous variables will also be described by absolute value and as change from baseline per analysis time point, if applicable.

Analyses will be performed for the total study population (overall analysis) and separately by indication or relevant subgroups (e.g., age, sex, GH pretreated and treatment-naive patients), if patient numbers are sufficient. Pretreated patients are here defined as patients already on treatment when enrolled in the study, whereas treatment-naive patients will be patients newly starting GHT.

The Statistical Analysis Plan (SAP) will specify details of the analysis.

### Coding

Concomitant medication will be coded using the WHO Drug Dictionary and tabulated by ATC term. Medical history, diseases and AEs will be coded by MedDRA in its current version [19], and/or by International Classification of Diseases (ICD).

All statistical details including calculated variables and proposed format and content of tables will be detailed in the SAP, which will be finalized before study database lock.

### Dissemination and communication of results

The information obtained during the conduct of this registry is considered confidential.

However, it is the explicit aim to publish the design, methods, results and insights derived from the registry.

The results of this registry are intended to be published in a series of articles in peer-reviewed journals and as abstracts/presentations at medical congresses under the oversight of the sponsor. Current guidelines and recommendation on good publication practice will be followed (e.g. STROBE [20]). No individual investigator may publish on the results of this study, or their own patients, without prior approval from the sponsor.

The site physicians will not disseminate any publication or release pertaining to the registry and/or results of the registry without consultation of the Sponsor and the Steering committee. Different parts of the registry may be published separately.

## Results

At the present time, there are no results available.

## Discussion

The INSIGHTS-GHT registry aims to document at least 1500 patients who receive treatment with GH. It has been designed to further the clinical understanding of this various indications that require the administration of GH, in a broad patient population across all age groups. The end goal of this registry is the synthesis, publication and dissemination of real-world evidence from the collected data, which can be used to improve both the understanding and clinical management of conditions that require GH. No other national registry on GH preparations is ongoing in Germany.

The registry documents children and adults in one joint registry, includes, at present, patients in Germany and allows documentation of patients on all approved somatropin and other growth hormone preparations. Once available, also the new long-acting GH preparations will be documented. Of note, INSIGHTS-GHT will allow to describe the transition of patients from adolescence to adulthood (with focus on treatment and final height), to describe switches between somatotropin preparations, to perform responder analyses, and to analyse differences and similarities of somatropin utilization (by age group, sex, setting, and PRO instrument).

A fundamental limitation of the INSIGHTS-GHT registry could be due to the different institutions involved in the registry and their recording of the collected auxological, laboratory chemistry, and radiological routine monitoring data. Because the registry is not a clinical trial, there is no mandatory data set to be collected other than informed consent and demographic data. To mitigate this limitation, the Steering Committee has created a core data set based on existing disease guidelines that would be desirable to use. This core data set will help standardize information across all institutions participating in the registry. In addition, we consider the limited applicability of the FAIR principles (discoverability, accessibility, interoperability, and Reuse of Digital Resources) [21] in their entirety to this clinical trial of GH use in the various approved indications as a potential limitation. This is due to existing privacy and confidentiality restrictions on the use and sharing of patient-level data. Although the patient-level data will not be publicly available, the results of aggregate data from the INSIGHTS-GHT Registry will be published in peer-reviewed journals. In addition, because the INSIGHTS-GHT Registry is registered under the BfArM No. NIS7492 and DRKS Registry DRKS00027394, the information is publicly available and appropriately qualified individuals may request access to the registry for research purposes.

## Conclusion

The study was initiated by investigators (see authors). The legal sponsor is the GWT-TUD GmbH in Dresden. The study entered the field phase in February 2022, and has enrolled 770 patients to date (08 May 2023). The field (documentation) phase of the study is planned until (at least) December 2024. The study is progressing as planned. So far, 23 centres are contributing patient data and the number is steadily increasing. The good progress is remarkable because there are currently several new PASS studies documenting long-acting preparations, so there are competing study projects in the centres.

## ICD and ORPHANET codes of diseases

**Table:** 

Disease	ICD-10	ORPHANET codes
Growth hormone deficiency (GHD)	E23.0	631, 91354, 467, 90695
		231662, 231671, 231679, 231692
Ullrich-Turner syndrome	Q96.0–96.9	881
Noonan syndrome	Q87.1	648
Prader–Willi syndrome	Q87.1	739
SHOX deficiency	Q87.1	not avaible (no rare disease)
Chronic renal failure	N17-N19	not avaible (no rare disease)

Patients with other conditions treated with GH are eligible for documentation

## Data Availability

Data sharing is not applicable to this article as datasets are being generated but have not been analysed during the current study.

## Abbreviations

AE: Adverse event
ATC: Anatomical therapeutic chemical (classification system)
BfArM: Bundesinstitut für Arzneimittel und Medizinprodukte
BIA: Bioelectronical impedance analysis
BMI: Body mass index
CRF/eCRF: Case report form/electronic case report form
CRI: Chronic renal insufficiency
DXA: Dual-energy X-ray absorptiometry
EDC: Electronic data capture
GH: Growth hormone
GHD: Growth hormone deficiency
GHT: Growth hormone treatment
HDL: High density lipoprotein
HV: Height velocity
IGF-I: Insulin-like growth factor 1
IGFBP-3: Insulin-like growth factor-binding protein 3
IHT: Insulin hypoglycemia test
INN: International nonproprietary name
LDL: Low density lipoprotein
MedDRA: Medical dictionary for regulatory activities
PWS: Prader-Willi syndrome
QoL: Quality of life
SAP: Statistical analysis plan
SDS: Standard deviation score
SF-12: Short form 12
SGA: Short for gestational age
SmPC: Summary of product characteristics
WHO: World Health Organization

## Footnotes

1 denotes parameters relevant only for patients with GH treatment initiation during childhood or adolescence.
